# Identification of a unique stress response state of T cells-related gene signature in patients with gastric cancer

**DOI:** 10.18632/aging.205895

**Published:** 2024-06-06

**Authors:** Qin Yang, Xin Li, Weiyuan Zhu

**Affiliations:** 1 Puai Medical College, Shaoyang University, The First Affiliated Hospital of Shaoyang University, Shaoyang, Hunan, China; 2 Department of Immunology, School of Basic Medicine, Central South University, Changsha, Hunan, China

**Keywords:** gastric cancer, gene signature, tumor microenvironment, stress response state of T cells, tumor immunity

## Abstract

Gastric cancer (GC), the third most lethal cancer worldwide, is often diagnosed at an advanced stage, leaving limited therapeutic options. Given the diverse outcomes among GC patients with similar AJCC/UICC-TNM characteristics, there is a pressing need for more reliable prognostic tools. Recent advances in targeted therapy and immunotherapy have underscored this necessity. In this context, our study focused on a novel stress response state of T cells, termed T_STR_, identified across multiple cancers, which is associated with resistance to immunotherapy. We aimed to develop a predictive gene signature for the T_STR_ phenotype within the tumor microenvironment (TME) of GC patients. By categorizing GC patients into high and low T_STR_ groups based on the infiltration states of TME T_STR_ cells, we observed significant differences in clinical prognosis and characteristics between the groups. Through a multi-step bioinformatics approach, we established an eight-gene signature based on genes differentially expressed between these groups. We conducted functional validations for the signature gene *PDGFRL* in GC cells. This gene signature effectively stratifies GC patients into high and low-risk categories, demonstrating robustness in predicting clinical outcomes. Furthermore, these risk groups exhibited distinct immune profiles, somatic mutations, and drug susceptibilities, highlighting the potential of our gene signature to enhance personalized treatment strategies in clinical practice.

## INTRODUCTION

Globally, gastric cancer (GC) continues to have a high incidence and mortality rate, with more than 1 million new cases diagnosed annually and approximately 784,000 deaths reported in 2018 [1, 2]. Although there has been a steady decline in the incidence and mortality rates of gastric cancer in most countries over the past few decades, the number of cases is expected to rise due to aging populations [1]. Chemotherapy, targeted therapy, and immunotherapy are the most common treatment options for patients with unresectable GC. However, the effectiveness of these therapies can be limited in specific patient groups. Advances in molecular analysis have enhanced our understanding of GC’s diverse characteristics, paving the way for the classification of patients into distinct groups and guiding the development of tailored treatments [1–3]. However, in the real world, only a few molecular classifications—including microsatellite instability (MSI), Epstein-Barr virus-positive (EBV+), genomically stable, and chromosomal instability groups—are currently utilized to support clinical decision-making regarding prognosis and therapy response in GC [1, 4], highlighting a significant need for novel predictive tools that can classify GC patients based on likely outcomes.

Due to the heterogeneous subpopulations and states of tumor-infiltrating T cells, some groups demonstrate anticancer efficacy in various immunotherapies, while others have the opposite effect. Recently, Yanshuo Chu and colleagues have identified a unique stress response state of T cells, termed T_STR_, for the first time, which adds a new layer of complexity to our understanding of tumor immunology [5]. T_STR_ cells are low in abundance or undetectable in healthy tissues. T_STR_ cells are found *in situ* of the tumor microenvironment (TME) across cancers and mainly detectable in lymphocyte aggregates or potential tertiary lymphoid structures in cancer beds or surrounding cancer edges [5]. CD4+ and CD8+ T_STR_ cells have been shown to be clinically relevant in cancer treatment. Particularly in patients undergoing anti-PD1/PDL1 therapy, these cells are highly enriched in cancers that do not respond to treatment, both before and especially after therapy [5]. In recent years, the increasing knowledge of TME significance has shifted cancer research model from a cancer centricity to one that considers the TME as a whole [6]. The composition of the TME includes a variety of cellular and non-cellular components that collectively influence cancer initiation, progression, and prognosis. Tumor-infiltrating T cells, a key element within the TME, are not only used to stratify patients but are also considered a promising target for cancer treatment. [7, 8]. Analyzing the molecular characteristics and clinical relevance of T_STR_ cells in the TME will enhance our understanding of their role in gastric cancer (GC). Furthermore, developing a T_STR_-based prognostic tool will be beneficial for categorizing patients into distinct risk groups. This stratification can guide critical decisions in clinical management, including determining the appropriateness of treatment options. This study aims to establish a prognostic gene signature associated with the T_STR_ phenotype within the TME of GC patients. Initially, we examined the status of CD4+ and CD8+ T_STR_ cells in GC, ultimately focusing on CD8+ T_STR_ cells for detailed multi-step bioinformatics analysis. We categorized GC patients from The Cancer Genome Atlas (TCGA) cohort into high and low T_STR_ groups based on the infiltration of CD8+ T_STR_ cells within the TME. These groups exhibited significantly different clinical prognoses and characteristics, underscoring the potential of T_STR_-based stratification in understanding and treating gastric cancer. We then established and validated an eight-gene signature derived from the differentially expressed genes (DEGs) between the two TSTR groups. This gene signature effectively stratified GC patients into high and low risk categories, demonstrating reliability and robustness in prognostic predictions. We further validated the biological functions of a key signature gene, PDGFRL, through experimental studies in GC cells. Finally, we analyzed the differences in somatic mutation profiles, immune responses, and drug sensitivities between the high and low risk groups, highlighting their distinct clinical and molecular characteristics.

## MATERIALS AND METHODS

### Data download and preprocessing

We obtained gene expression profiles, clinical features, and single nucleotide variant data for the TCGA-STAD cohort using the “TCGAbiolinks” package [9]. As transcript per million data are more comparable to microarray data [10], we converted the FPKM-formatted expression matrix to transcript per million format for subsequent analyses. Expression profiles and clinical data from three additional external cohorts (GSE15459, GSE26899 and GSE29272) were collected through queries of the Gene Expression Omnibus database (https://www.ncbi.nlm.nih.gov/geo/). Single-cell RNA sequencing data were obtained from the TISCH2 database (http://tisch.comp-genomics.org/) [11]. The markers of CD4+ T_STR_ cells and CD8+ T_STR_ cells were based on a previous study [5].

### Functional enrichment analysis

Differential expression analysis between tumor and normal tissues was performed using the limma package [12] to identify genes with fold changes. Benjamin-Hochberg method was used to correct P-values. Genes with |log2FC| > 0.585 and adjusted P-value < 0.05 were considered DEGs. Gene Set Enrichment Analysis (GSEA) was then applied to examine enrichment within specific gene sets. Gene Ontology (GO) and Kyoto Encyclopedia of Genes and Genomes (KEGG) enrichment analyses were conducted using the clusterProfiler package [13]. The protein-protein interaction (PPI) network was retrieved from the STRING database (http://string-db.org/), which contains both predicted and experimentally determined functional associations between proteins. The nodes were analyzed and visualized using Cytoscape software. A functionally grouped network was constructed based on GO terms using the ClueGO plugin for Cytoscape, which allows integration of GO annotations with PPI networks.

### Identification of CD8+ T_STR_ cell-related groups in GC patients

Single-sample gene set enrichment analysis (ssGSEA) algorithm was performed to quantify the infiltration levels of CD8+ T_STR_ cells in each GC patient. Referring to similar research [14, 15], we conducted the survminer package to determine the optimal cutpoints. Kaplan-Meier survival curves were plotted using the product-limit estimates method to compare clinical outcomes between the two groups, with *P*-value determined by the log-rank test. Principal component analysis (PCA) was applied to validate the distinct distributions between the two groups.

### Establishment and validation of an eight-gene signature associated with CD8+ T_STR_ cells

We performed the limma package to identify differentially expressed genes (DEGs) between samples with high CD8+ T_STR_ cell levels and low CD8+ TSTR cell levels based on a threshold of (|log2FC|) > 0.585 and an adjusted P-value < 0.05 (corrected by Benjamin-Hochberg method). Univariate Cox regression analysis was then used to identify any DEGs associated with prognosis. Finally, the least absolute shrinkage and selection operator (LASSO) algorithm was employed to construct an eight-gene signature associated with CD8+ T_STR_ cells, using the following calculation:


$$Risk\;score=\sum_{i=1}^{n}Coef_{i}\times Exp_{i}$$

Based on the median, GC patients were dichotomized into high-risk and low-risk groups. Univariate cox and multivariable cox analyses were carried out to determine if the risk score or other clinical features (including age, gender, stage, recurrence, and other pathological parameters) were independent prognostic factors in GC patients. Subsequently, a nomogram signature was established by combining the risk score with other independent prognostic factors, and the corresponding receiver operating characteristic (ROC) and calibration curves for 1-, 3-, 5-year were plotted to confirm the predictive accuracy.

### Tumor microenvironment (TME) analysis

The ESTIMATE algorithm [16] was used to evaluate the levels of immune and stromal cell infiltration in GC patients. Additionally, the MCP-counter algorithm [17] assessed the levels of specific immune and stromal cell types present within the tumor tissues. To validate the differences in cancer-associated fibroblasts (CAFs) between the high-risk and low risk patient groups stratified by the eight-gene signature, the EPIC algorithm [18] was applied.

### Single-cell RNA sequencing analysis

The Seurat package was used to analyze the scRNA-seq dataset GSE134520. Canonical markers were used to define nine main cell types of present: CD8+ T cells, dendritic cells, fibroblasts, glandular mucous cells, malignant cells, macrophages, smooth muscle cells, pit mucous cells, and plasma cells. The AddModuleScore procedure in Seurat was then applied to estimate expression scores of the previously developed eight-gene signature across each of these different cell populations.

### Analysis of immunotherapy efficacy and anti-cancer drug sensitivity

The maftools package [19] was used to count the frequencies of mutated genes and calculate the tumor mutation burden (TMB) values for each GC patient. The TIDE online tool (http://tide.dfci.harvard.edu/) was utilized to predict the response to immunotherapy in TCGA-STAD cohort [20]. Additionally, the predictive ability of the eight-gene signature was validated in two immunotherapy cohorts (GSE176307 and IMvigor210). The GSE176307 cohort includes 90 patients diagnosed with urothelial carcinoma who were treated with immunotherapies targeting the PD-1/PD-L1 axis. The IMvigor210 cohort is composed of 348 patients with urothelial cancer who were treated with atezolizumab, a monoclonal antibody specifically designed to bind to PD-L1 and block its interactions with PD-1 and B7.1 receptors. To guide personalized treatment for GC patients, the oncoPredict package [21] was implemented to predict the sensitivity to different anti-cancer drugs for the two risk groups.

### Cells and cell culture

Normal gastric cells GES-1 and GC cells AGS, MKN-45, SUN-1 and HGC-27 were purchased from the Cell Bank of Type Culture Collection of the Chinese Academy of Sciences, Shanghai Institute of Cell Biology. All cell types were cultured in 1640 +10% FBS and incubated at 37° C, 5% CO_2_ concentration and an appropriate humidified atmosphere.

### Antibodies, siRNAs and reagents

Rabbit PDGFRL antibody was purchased from Proteintech Co., Ltd (Wuhan, China). Mouse β-actin antibody was purchased from ProMab Biotechnologies Co., Ltd (Richmond, USA). PDGFRL siRNA and GP-transfect-Mate transfection kit was purchased from Shanghai GenePharma Co., Ltd (Shanghai, China). siRNAs targeting PDGFRL was transfected into AGS cells following the manufacturer’s instruction. siRNAs targeting PDGFRL (siPDGFRL-1#: 5’GCCAACACCUUCCCAAGAATT3’, siPDGFRL-2#: 5’GCGUAUCUGGACACCUUUATT’ and siPDGFRL-3#: 5’GCCAACACCUUCCCAAGAATT 3’).

### RT-qPCR and western blot

RT-qPCR was carried out as we previously described [7, 22, 23]. RT-qPCR primers for PDGFRL (forward: 5’GACGACATCAGTGTGCTCTGCA3’, revers: 5’CCAAGTGTCTTGGATCGTCACAG3’). Cells were lysed with RIPA buffer that contained a protease or phosphatase inhibitor mixture. 20 μg proteins were separated by SDS-PAGE gels, transferred onto nitrocellulose membranes, and incubated with antibodies.

### MTT, wound healing and transwell

MTT, wound healing and transwell experiments were also performed according to our previous protocol [7, 22, 23].

### Statistical analysis

The statistical analysis and data visualization were conducted using R software (v4.2.2) or Cytoscape software. Unless otherwise stated, a two-tailed Student’s t-test was utilized to compare differences between distinct CD8+ T_STR_/risk groups. Spearman rank sum test was used for correlation analysis. A *P*-value less than 0.05 was considered statistically significant.

### Availability of data and material

mRNA expression profile and follow-up information are downloaded from the TCGA (https://www.cancer.gov/about-nci/organization/ccg/research/structural-genomics/tcga) and GEO (http://www.ncbi.nlm.nih.gov/geo/) databases. Further results or code inquiries can be directed to the corresponding author.

## RESULTS

### Profile of T_STR_ cells-related genes

**Figure 1:**
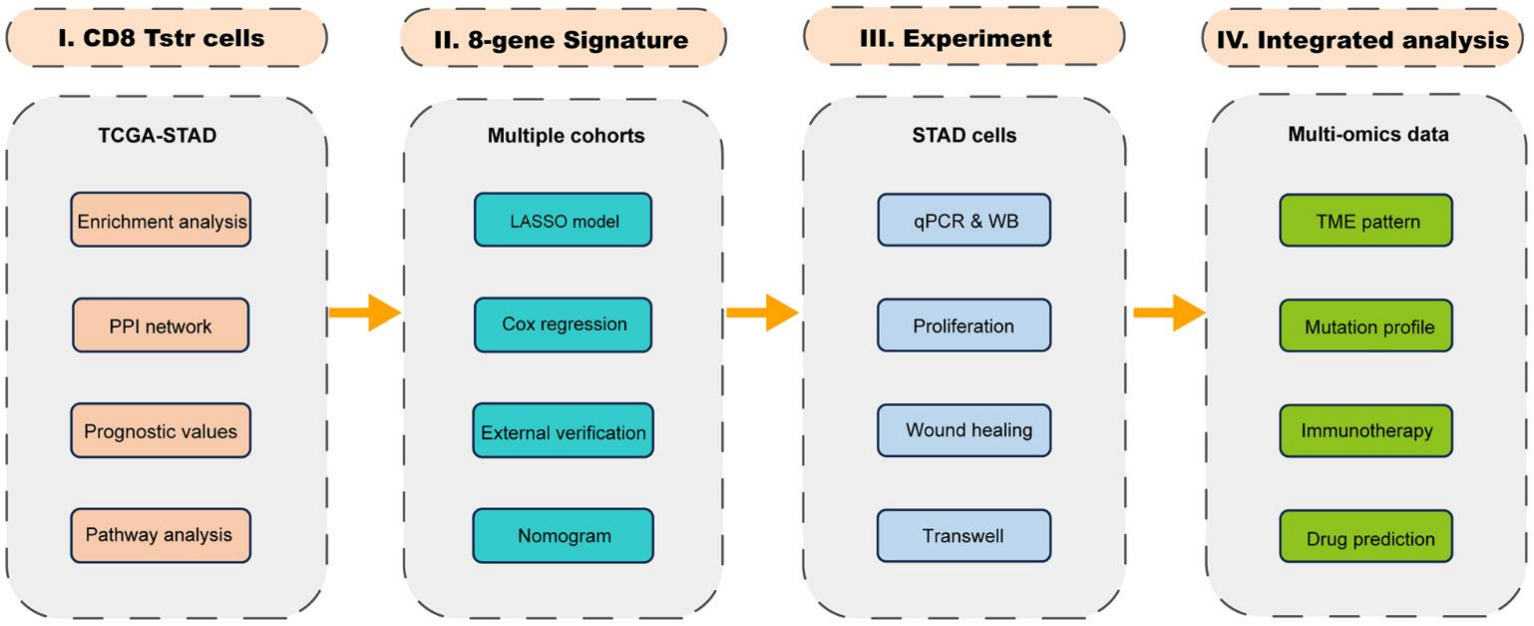
The flow chart.

**Figure 2:**
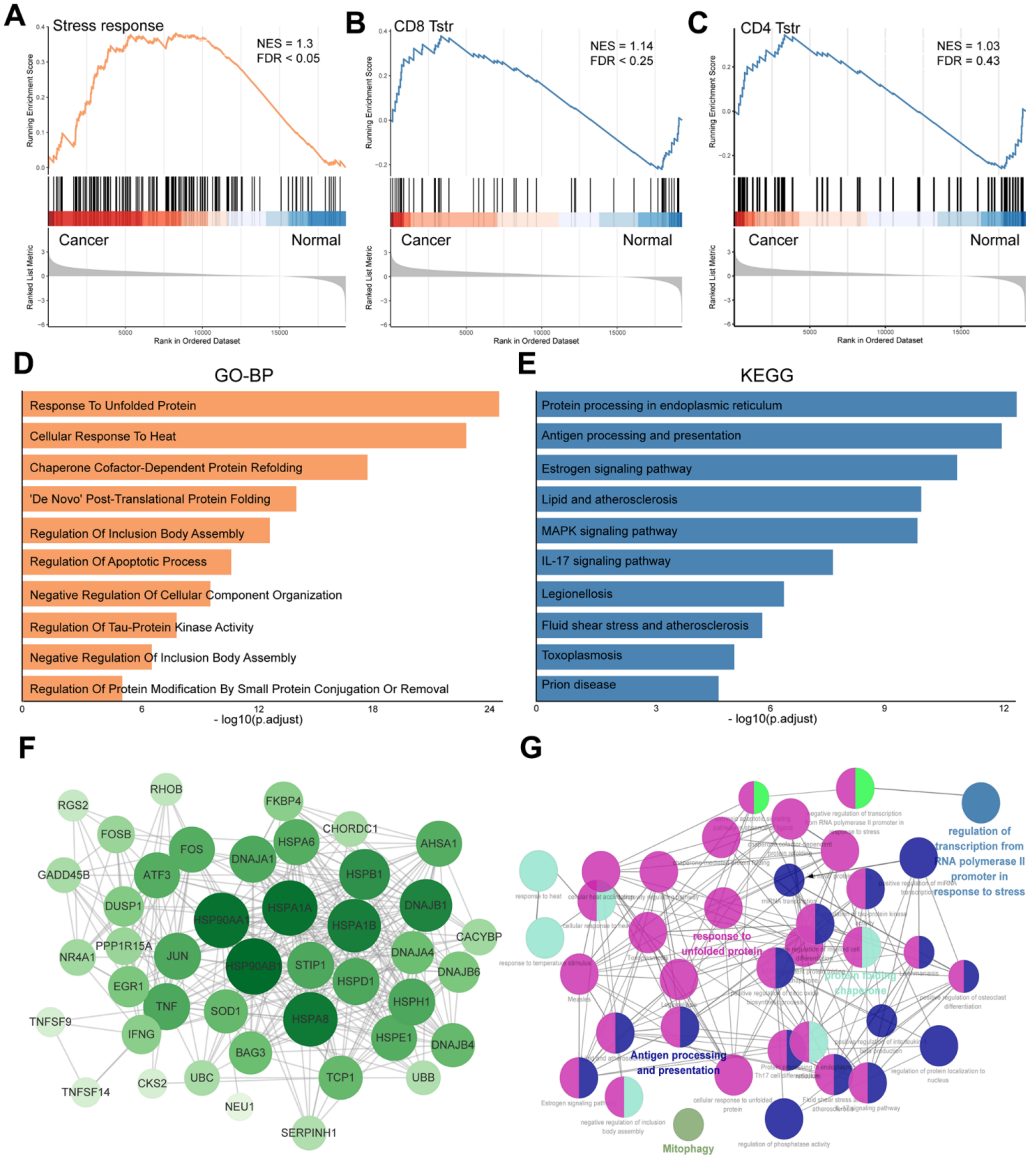
**Profile of T_STR_ cells-related heat shock genes in GC.** (**A**) GSEA enrichment analysis of T_STR_ cells-related heat shock genes. (**B**) GSEA enrichment analysis for CD8+ T_STR_ cells. FDR < 0.25 indicates significant. (**C**) GSEA enrichment analysis for CD4+ T_STR_ cells. FDR > 0.25 indicates not significant. (**D**) GO-BP enrichment analysis for CD8+ T_STR_ cells. (**E**) KEGG pathway enrichment analysis for CD8+ T_STR_ cells. (**F**) Protein-protein interaction network of top T_STR_ cells-related heat shock genes for CD8+ T_STR_ cells. (**G**) Potential biofunctional network associated with T_STR_ cells-related heat shock genes for CD8+ T_STR_ cells.

Figure 1 presents a flow chart of our study, which investigates T_STR_ cells—a unique stress response state of T cells characterized by the expression of heat shock genes [5]. To date, the T_STR_ status has not been explored in GC. To understand the profile of T_STR_ cells-related marker genes, including 163 genes, we conducted Gene Set Enrichment Analysis (GSEA), Gene Ontology Biological Processes (GO-BP), Kyoto Encyclopedia of Genes and Genomes (KEGG), and Protein-Protein Interaction (PPI) network analyses. Our GSEA results indicated a significant stress response in cancer tissues compared to normal tissues (Figure 2A). Additionally, we observed a significant enrichment of CD8+ T_STR_ cells in cancer tissues compared to normal tissues (Figure 2B), unlike CD4+ T_STR_ cells, which showed no significant enrichment (Figure 2C). Consequently, we focused subsequent analyses solely on CD8+ T_STR_ cells.

The GO-BP analysis identified key processes including response to unfolded protein, cellular response to heat, chaperone cofactor-dependent protein refolding, and De Novo post-translational protein folding (Figure 2D). The KEGG analysis highlighted enrichment in pathways such as protein processing in the endoplasmic reticulum, antigen processing and presentation, estrogen signaling, lipid metabolism and atherosclerosis, MAPK signaling pathway, and IL-17 signaling pathway (Figure 2E). We utilized the STRING platform and cytoHubba procedure to analyze the potential biological interactions and biofunctional networks among CD8+ T_STR_ cell-related genes, illustrated in Figure 2F, 2G, respectively.

### CD8+ T_STR_ cells function as a poor prognostic factor in GC

**Figure 3:**
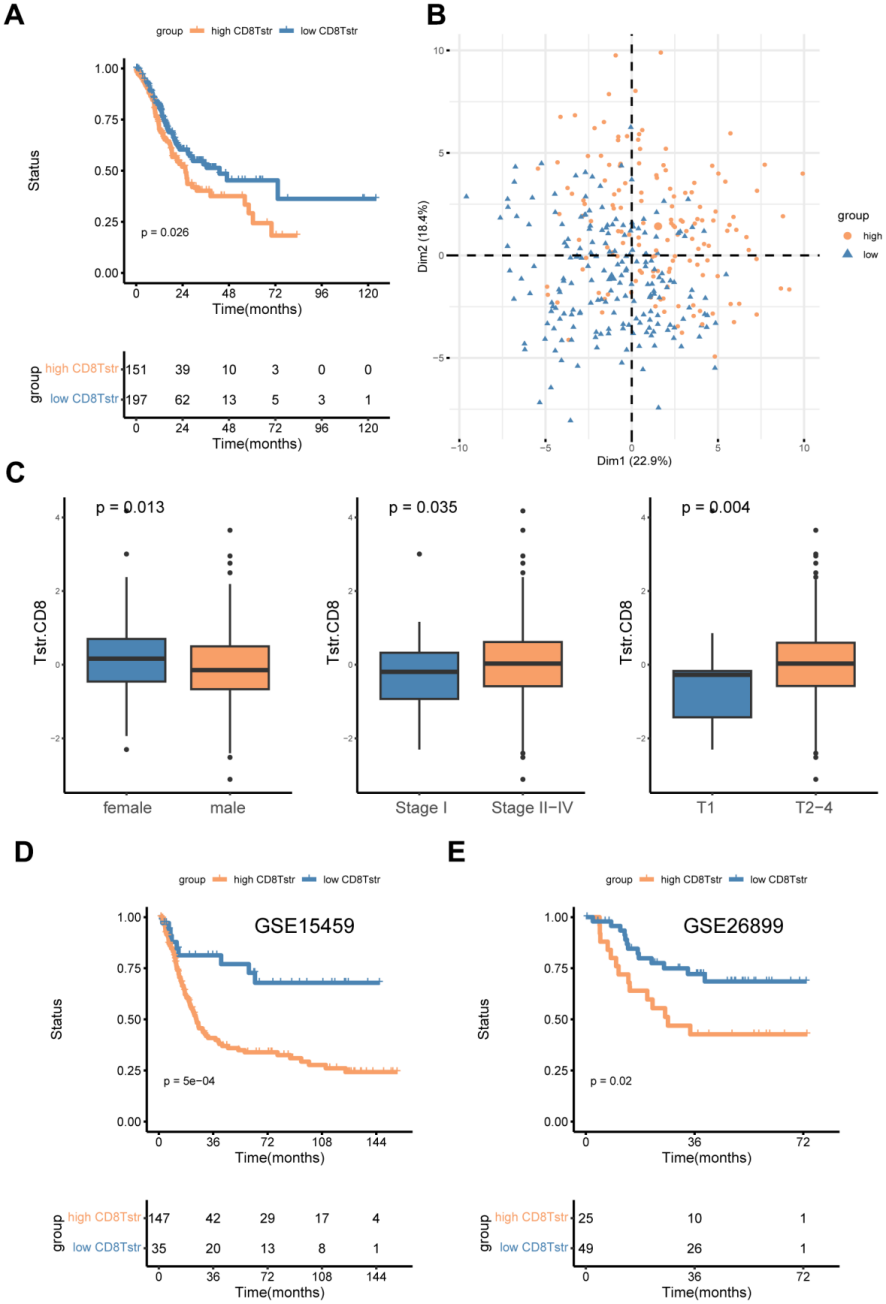
**High/low CD8+ T_STR_ groups classification.** (**A**) Kaplan-Meier survival curves for OS in the training cohort TCGA-GC. (**B**) PCA for the high/low CD8+ T_STR_ groups. (**C**) Infiltration of CD8+ T_STR_ cells according to gender (female/male), stage (I/II -IV) and T stage (1/2-4). (**D**) Kaplan-Meier survival curves for OS in the GC dataset GSE15459. (**E**) Kaplan-Meier survival curves for OS in the GC dataset GSE26899.

We employed the ssGSEA algorithm to assess the infiltration levels of CD8+ T_STR_ cells in each GC patient, classifying them into low and high CD8+ T_STR_ groups based on their infiltration scores. Our analysis revealed that patients in the low CD8+ T_STR_ group exhibited a significant survival advantage over those in the high CD8+ T_STR_ group within the TCGA-GC dataset (Figure 3A). A PCA plot further confirmed clear separation between the two groups (Figure 3B). We also evaluated the clinical relevance of this classification by gender (female/male), stage (I/II - IV), and T stage (1/2 - 4), finding significantly higher infiltration of CD8+ T_STR_ cells in females and significantly lower infiltration in early-stage (I and T stage 1) patients compared to their counterparts (Figure 3C). Additionally, the low CD8+ T_STR_ group demonstrated significantly longer overall survival (OS) than the high CD8+ T_STR_ group in two validation datasets from GEO-GC datasets (Figure 3D, 3E).

### Enrichment analyses of the CD8+ T_STR_ cells-related DEGs

**Figure 4:**
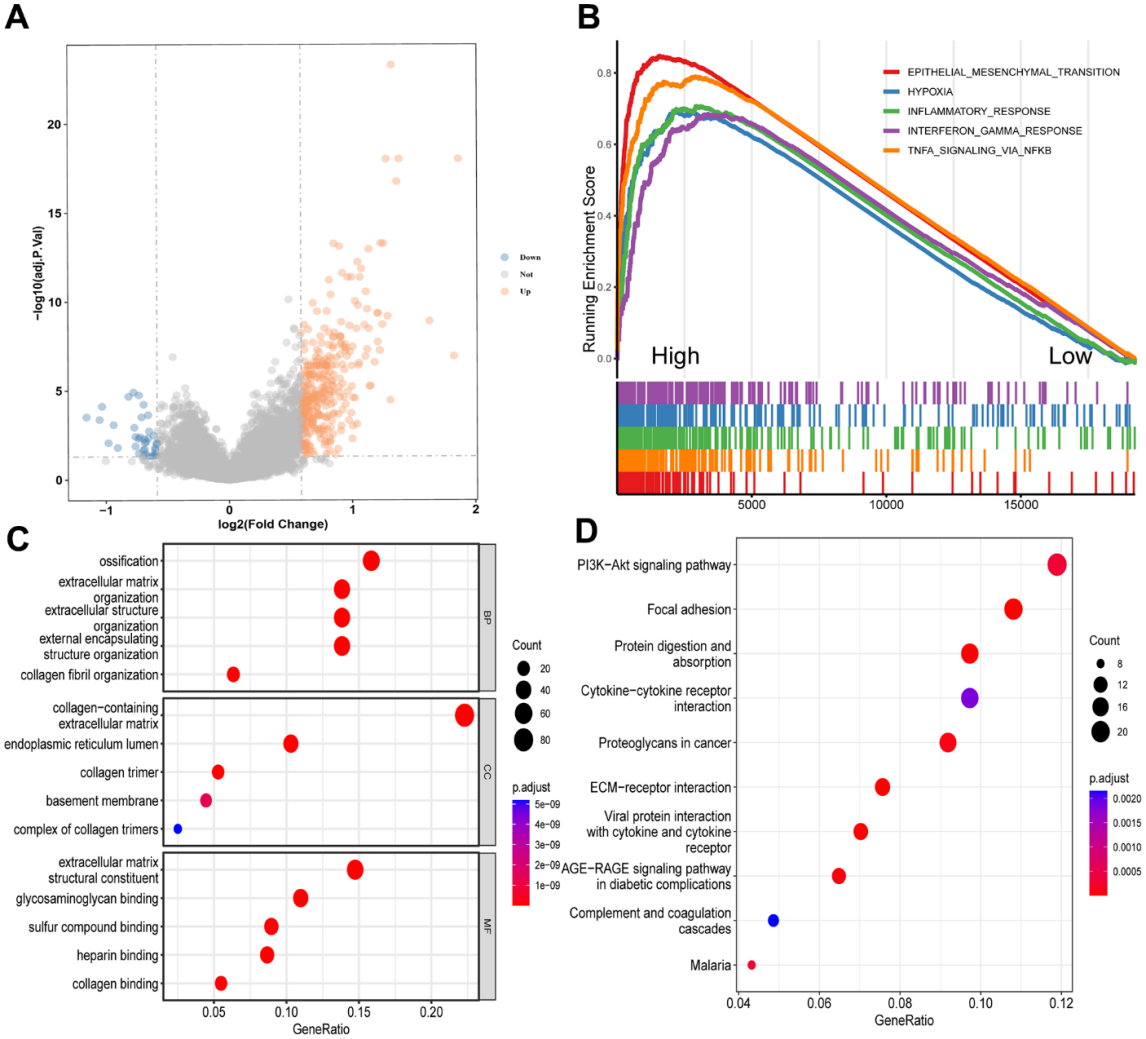
**Enrichment analyses of DEGs between the high and low CD8+ T_STR_ groups.** (**A**) Volcano plot showing the DEGs. The horizontal dashed line represents p.adjust value = 0.05. (**B**) GSEA analysis of the top 5 gene hallmarks. (**C**) GO analyses for BP, CC and MF. (**D**) KEGG analysis.

To investigate the association between CD8+ T_STR_ cell infiltration and biological behaviors in gastric cancer, we first identified DEGs between the high and low CD8+ T_STR_ groups. A total of 363 DEGs were found, with 326 being upregulated and 37 downregulated (Figure 4A). Subsequently, we conducted a GSEA using functional annotations from the TCGA-GC dataset. The top five hallmark gene sets identified were epithelial-mesenchymal transition, hypoxia, inflammatory response, interferon gamma response, and TNF-α signaling via NF-κB (Figure 4B). Further functional insights were gained through GO and KEGG analyses on the DEGs. The GO-BP primarily included ossification, organization of extracellular matrix and structures, and collagen fibril organization (Figure 4C). The GO Cellular Component (GO-CC) analysis highlighted enrichment in structures such as the collagen-containing extracellular matrix and endoplasmic reticulum lumen (Figure 4C). The most prominent GO Molecular Functions (GO-MF) involved binding activities related to the extracellular matrix, including glycosaminoglycan, sulfur compound, heparin, and collagen binding (Figure 4C). KEGG pathway enrichment analysis revealed significant pathways such as PI3K-Akt signaling, focal adhesion, protein digestion and absorption, cytokine-cytokine receptor interaction, proteoglycans in cancer, and ECM-receptor interaction (Figure 4D).

### Identification of a CD8+ T_STR_-related eight-gene signature

**Figure 5:**
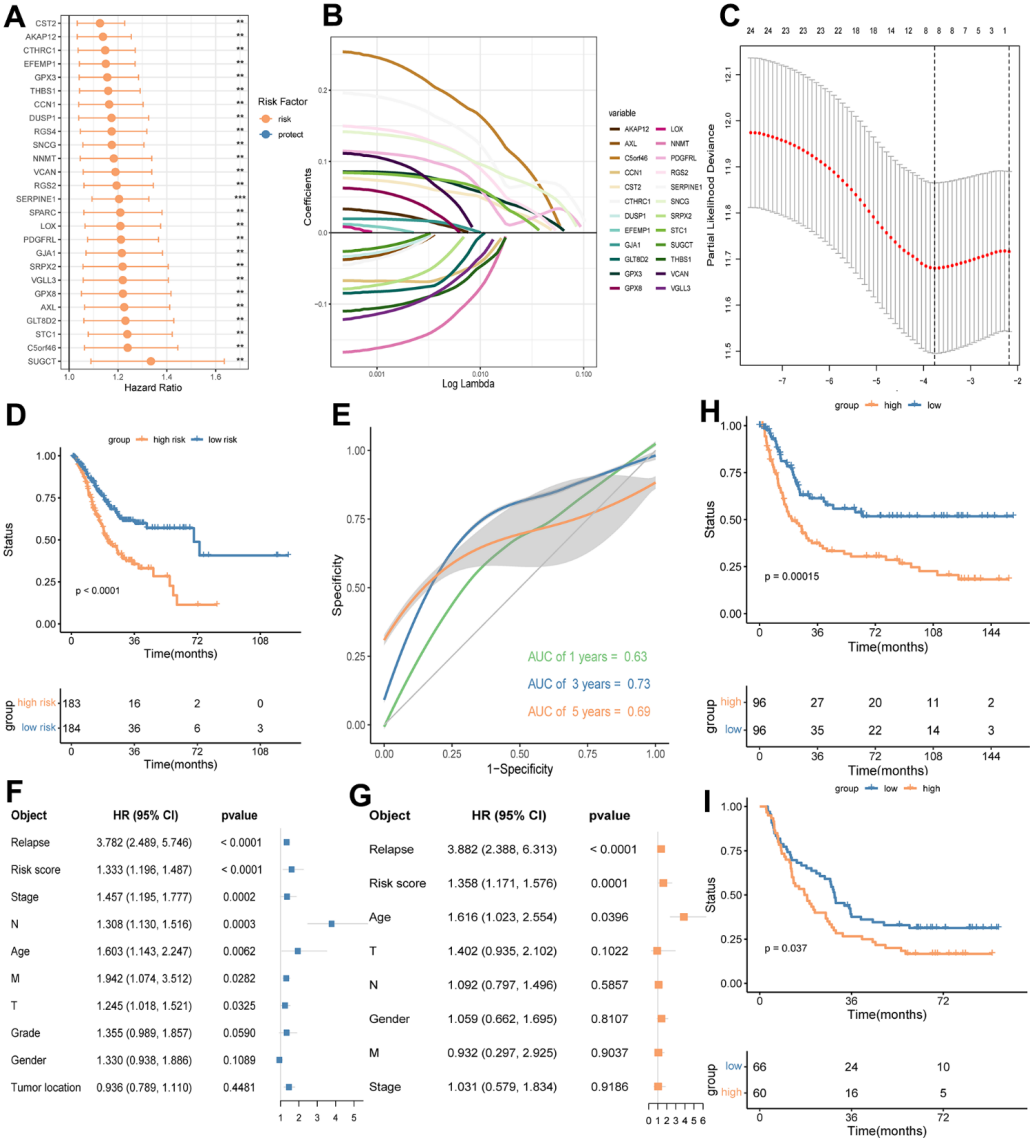
**Identifying a prognostic eight-gene signature base on DEGs between the high and low CD8+ T_STR_ groups in GC.** (**A**) HR forest plot of prognostic genes identified by a multivariate cox analysis. HR > 1 indicates risk factors. HR < 1 indicates protection factors. (**B**) Partial likelihood deviance coefficient profiles. (**C**) LASSO of the DEGs. Eight genes with the strongest predicting power are identified. (**D**) OS analysis for the high and low risk groups using the TCGA-GC training cohort. (**E**) ROC curves for 1, 3 and 5 years survival predictions using the eight-gene signature. (**F**) Multivariate cox regression analysis shows risk score of the eight-gene signature and other prognosis-related clinic factors. (**G**) Univariate cox regression analysis shows risk score of the eight-gene signature and other prognosis-related clinic factors. (**H**) OS analysis for the high and low risk groups using GSE15459-GC cohort. (**I**) OS analysis for the high and low risk groups using GSE29272-GC cohort.

To assess the prognostic value of CD8+ TSTR-related DEGs, we used the TCGA-GC dataset as a training cohort. Initial univariate Cox regression analysis identified genes with significant prognostic value, and their hazard ratios (HR) are displayed in Figure 5A. Subsequently, we applied the LASSO algorithm to construct a prognostic eight-gene signature, detailed in Figure 5B, 5C. The risk score for each GC patient was calculated using the following formula: Risk Score = 0.0650 * SERPINE1 + 0.0411 * RGS2 + 0.0138 * PDGFRL + 0.0248 * STC1 + 0.103 * C5orf46 + 0.0271 * CST2 + 0.0372 * GPX3 + 0.0737 * SNCG. Patients were then divided into high and low risk groups based on the median risk score. Kaplan-Meier survival analysis revealed that the low-risk group had significantly better overall OS than the high-risk group (Figure 5D). ROC curves confirmed the good sensitivity and specificity of the eight-gene signature in classifying GC patients (Figure 5E).

To determine the independence of the eight-gene signature, we conducted univariate and multivariate Cox regression analyses using the risk score and other prognostic factors—such as relapse, stage, N stage, age, M stage, T stage, grade, gender, and tumor location—as covariates. The results confirmed that our gene signature is an independent risk factor (Figure 5F, 5G). Validation of the eight-gene signature in external GC cohorts from GEO datasets GSE15459 and GSE29272 showed that the high-risk group consistently exhibited worse outcomes than the low-risk group (Figure 5H, 5I). These findings collectively demonstrate that our eight-gene signature effectively classifies GC patients and offers robust prognostic utility.

### Functional validation of the signature gene PDGFRL

The biological functions of the eight signature genes have been explored in GC, except for PDGFRL, which is identified as a risk factor in this study. Currently, our understanding of PDGFRL in cancer biology is limited. Previous research has only examined its functions in chondrocytic HCS-2/8 cells and breast cancer-derived MDA-MB-231 cells. Intriguingly, PDGFRL exhibits cell type-dependent roles, functioning as an oncogene in chondrocytes and as a tumor suppressor gene in breast cancer cells [24].

**Figure 6:**
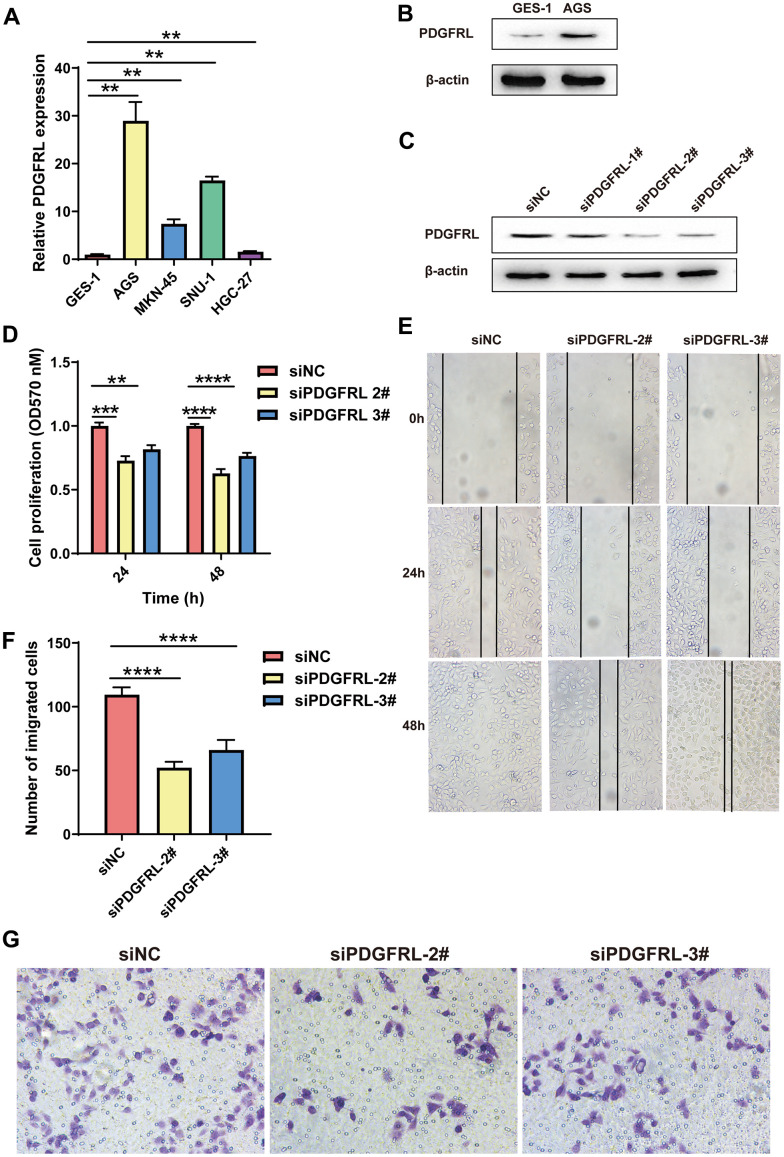
**Functional validation for PDGFRL in GC cells.** (**A**) PDGFRL mRNA expressions in normal gastric cells GES-1 and GC cells AGS, MKN-45, SUN-1 and HGC-27. (**B**) PDGFRL protein expressions in GES-1 and AGS cells. (**C**) AGS cells were transiently transfected with three different PDGFRL siRNAs (siPDGFRL-1#, siPDGFRL-2# and siPDGFRL-3#) and their negative control (siNC). Western blot was used to measure PDGFRL expressions. β-actin was used as loading control. MTT assay (**D**), wound healing assay (**E**) and transwell assay (**F**) were used to measure proliferation, migration and invasion of AGS cells, respectively. Representative images of the transwell assay were shown (**G**). The data were presented as the mean ± standard deviation. *** *P* < 0.01, *** *P* < 0.001 and **** *P* < 0.0001.

In this study, we assessed PDGFRL mRNA expression through qPCR experiments in normal gastric cells GES-1 and several GC cell lines (AGS, MKN-45, SUN-1, and HGC-27). As shown in Figure 6A, PDGFRL mRNA levels were significantly elevated in all GC cell lines compared to normal gastric cells, with AGS cells exhibiting the highest expression levels. Similarly, PDGFRL protein expression was higher in AGS cells than in GES-1 cells (Figure 6B), prompting us to select AGS cells for further functional validation.

We transfected AGS cells with PDGFRL-targeting siRNAs (siPDGFRL-1#, siPDGFRL-2#, and siPDGFRL-3#), successfully achieving gene knockdown. siPDGFRL-2# and siPDGFRL-3# were identified as optimal siRNAs for subsequent experiments, including MTT, wound healing, and transwell assays (Figure 6C). Results from these assays showed significant reductions in cell proliferation (Figure 6D), migration (Figure 6E), and invasion (Figure 6F, 6G) in PDGFRL-silenced AGS cells compared to controls, suggesting that PDGFRL may act as a cancer promoter in this context. These findings align with its identified prognostic value in GC.

### Associations between the signature risk score and clinical parameters

**Figure 7:**
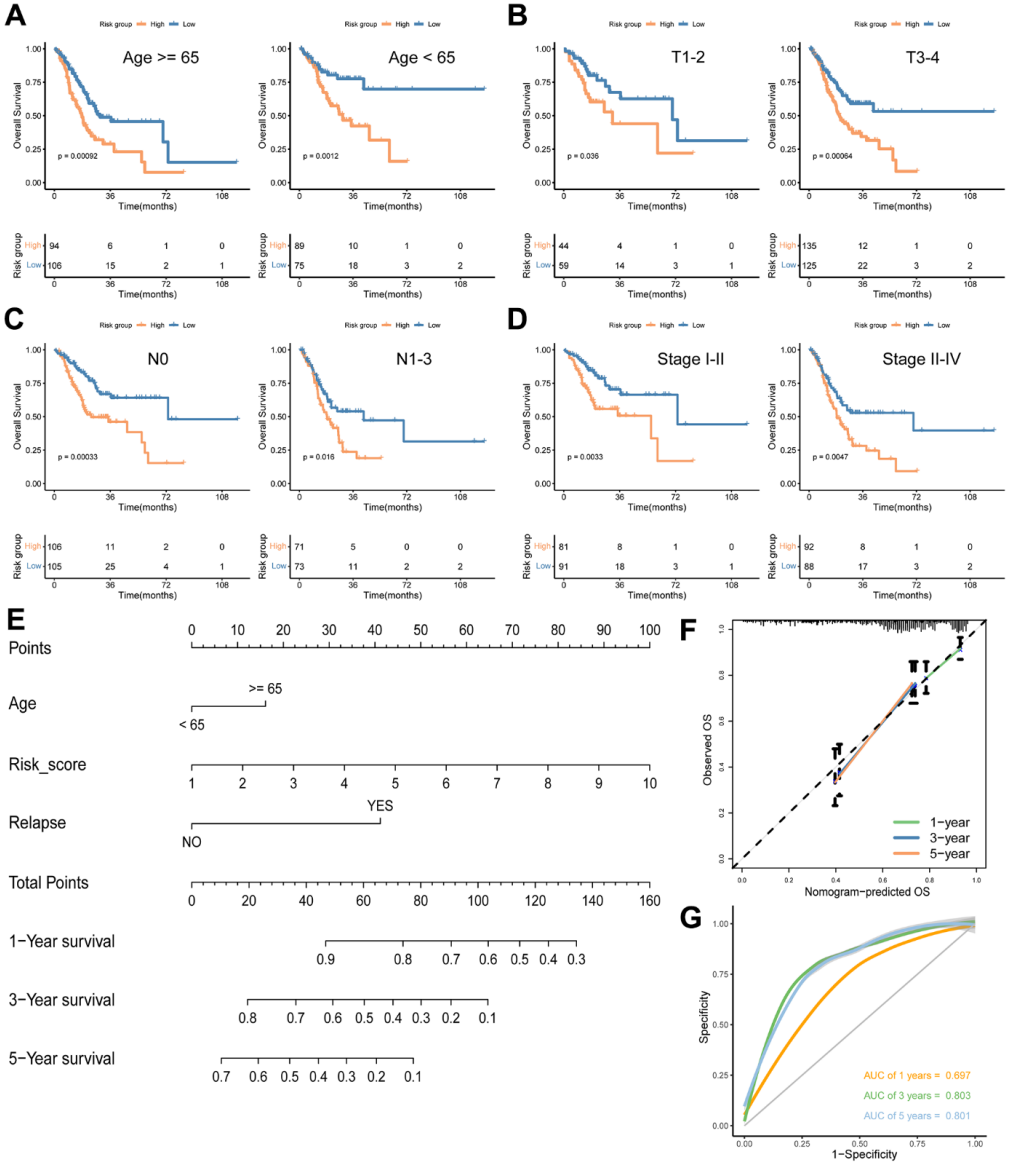
**Analyses of associations between the signature risk score and other clinical parameters.** Survival analyses according to age (**A**), T stage (**B**), N stage (**C**) and stage (**D**). (**E**) A nomogram including age, risk score and relapse for predicting 1-, 3-, and 5- year survival in GC. Calibration curves (**F**) and ROC curves (**G**) at 1-, 3-, and 5-year are used for determining the efficacy and reliability of the monogram.

To explore the relationship between the signature risk score and various clinical characteristics, we conducted survival analyses across different clinical parameters, including age (≥ 65/< 65) (Figure 7A), T stage (T1-2/T3-4) (Figure 7B), N stage (0/1-3) (Figure 7C) and stage (I-II/III-IV) (Figure 7D). In each category, patients in the low-risk group consistently demonstrated significantly longer survival times compared to those in the high-risk group (Figure 7A–7D). Additionally, we developed a nomogram for predicting OS at 1-, 3-, and 5-year intervals, incorporating age, risk score, and relapse (Figure 7E). The performance and reliability of this nomogram were validated using calibration curves (Figure 7F) and ROC curves (Figure 7G) for each time point. The results confirmed that the nomogram accurately predicts the OS of GC patients, showcasing its clinical utility (Figure 7E–7G).

### Analyses of the TME between different risk groups

**Figure 8:**
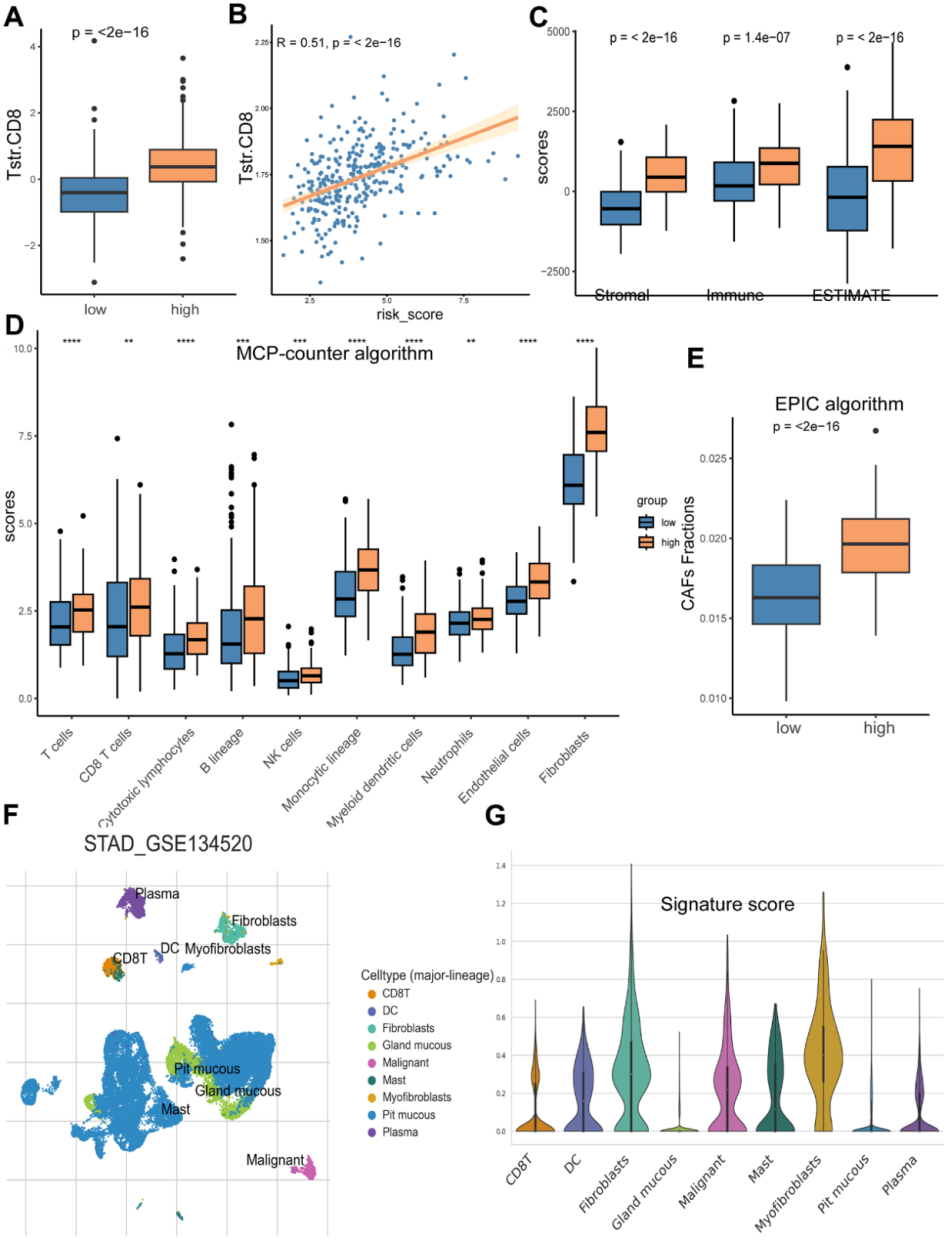
**Analyses of TME in the high/low risk groups.** (**A**) The high-risk group has a significantly higher CD8+ T_STR_ infiltration level than the low risk group. (**B**) The CD8+ T_STR_ infiltration level is significantly positively associated with the risk score. (**C**) The high-risk group has higher Stromal score, Immune score and ESTIMATE score than the low risk group. (**D**) Infiltration comparison of ten immune cell types between the two risk groups based on MCP-counter algorithm. (**E**) Comparison of CAFs infiltration between the high and low risk groups based on EPIC algorithm. (**F**) Distributions of nine cell types in GC tissues. (**G**) Scores of the eight signature genes in the nine cell types.

The TME plays a dynamic role in the oncogenesis of GC and significantly influences clinical outcomes. Our findings indicate that the TME of the high-risk group is markedly different from that of the low-risk group (Figure 8). Specifically, the high-risk group exhibited significantly higher levels of CD8+ T_STR_ cell infiltration compared to the low-risk group (Figure 8A), and there was a positive correlation between the risk score and CD8+ T_STR_ cell infiltration levels (Figure 8B). Furthermore, TME infiltration scores, including Stromal score, Immune score, and ESTIMATE score, were also higher in the high-risk group (Figure 8C).

We analyzed the infiltration levels of ten major TME cell types using the MCP-counter algorithm, finding significantly greater infiltration in all cell types within the high-risk group (Figure 8D), with fibroblasts showing the highest infiltration scores. Consistently, the high-risk group also demonstrated a higher level of CAFs based on the EPIC algorithm (Figure 8E). To delve deeper into the associations between the risk score from our eight-gene signature and specific cell types within the TME, we examined cell type composition using the single-cell dataset GSE134520. This analysis identified nine cell types, including CD8+ T cells, dendritic cells, fibroblasts, gland mucous cells, malignant cells, mast cells, myofibroblasts, pit mucous cells, and plasma cells, with their distribution shown in Figure 8F. Among these, fibroblasts had the highest signature risk score, followed by myofibroblasts and malignant cells (Figure 8G).

### Characterization of TMB and immunotherapy response

**Figure 9:**
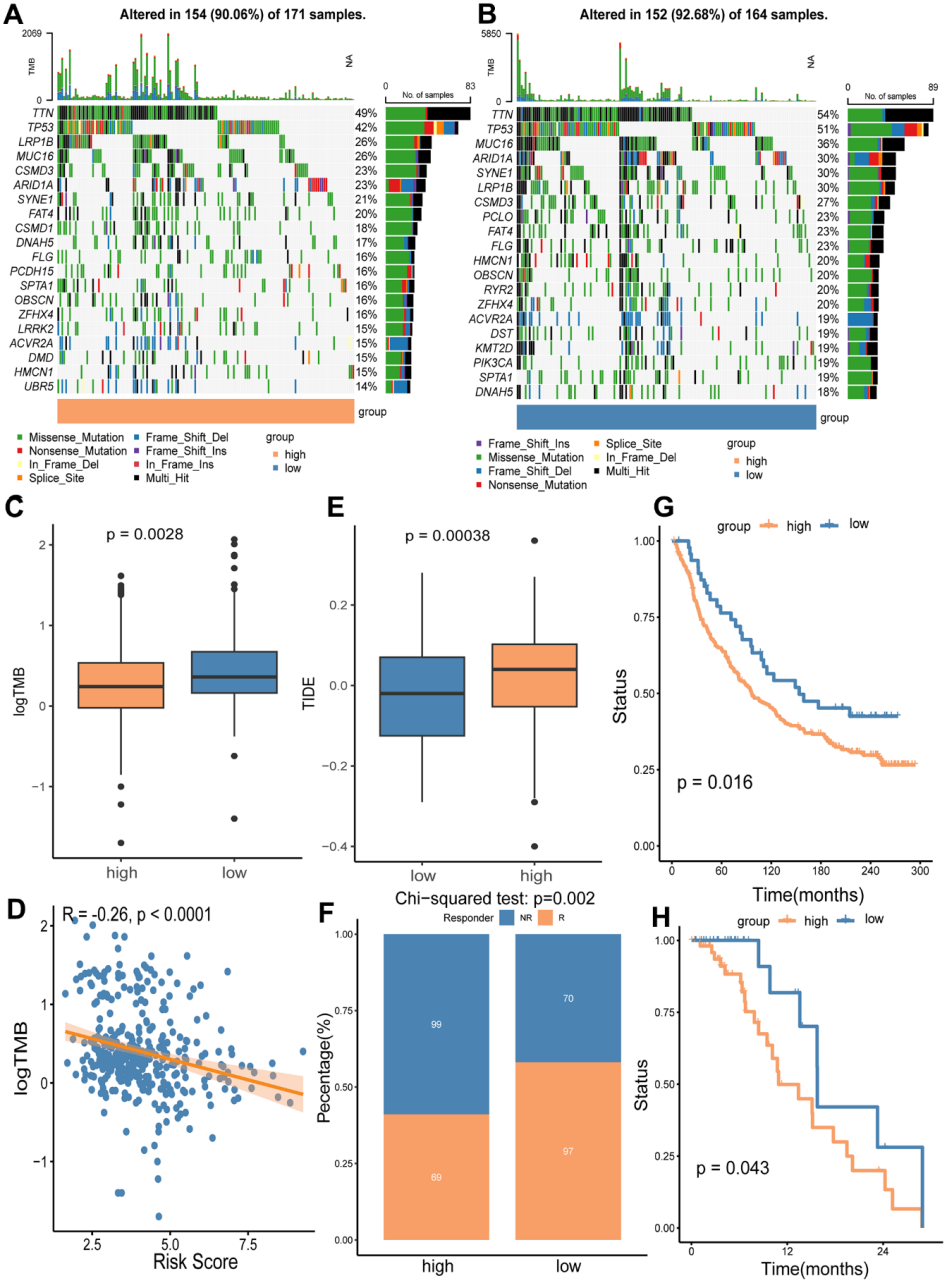
**Analyses of somatic mutation frequency and immunotherapy response in the high/low risk groups.** The waterfall plot shows the top 20 mutation genes in the high-risk group (**A**) and the low-risk group (**B**). (**C**) Differential TMB in the two risk groups. (**D**) The association analysis of TMB with the risk score. (**E**) Immunotherapy response prediction for the high and low risk groups using TIDE algorithm. (**F**) Comparison of non-responders (NR) and responders (R) between the two risk groups. (**G**) OS analysis of high and low risk groups in immunotherapy cohort IMvigor210. (**H**) OS analysis of the high and low risk groups in immunotherapy cohort GSE176307.

We utilized the maftools package to analyze mutation frequencies in two risk groups. Notably, the frequency of mutations in several key cancer-related genes was lower in the high-risk group compared to the low-risk group (TTN: 49% vs. 54%; TP53: 42% vs. 51%; MUC16: 26% vs. 36%; LRP1B: 26% vs. 30%) (Figure 9A, 9B). Additionally, a significant difference in TMB was observed between the groups (Figure 9C), with TMB negatively correlated with the risk score of our gene signature (Figure 9D). Given that higher TMB is associated with an increased capability to generate neo-antigens and potentially a better response to immunotherapy in various cancers, including GC [25, 26], we further investigated whether the signature risk scores could predict immunotherapeutic outcomes in GC patients. Our findings indicated that the high-risk group had a significantly higher Tumor Immune Dysfunction and Exclusion (TIDE) score (p = 0.0028) and a greater number of immunotherapy non-responders (99/168) compared to the low-risk group (70/167) (p = 0.002) (Figure 9E, 9F). Lastly, we analyzed OS using two immunotherapy cohorts (IMvigor210 and GSE176307) to examine differences between risk groups under immunotherapy. The results showed that the gene signature could distinguish GC patients well in the immunotherapy cohorts (Figure 9G, 9H).

### Drug sensitivity in different risk groups

**Figure 10:**
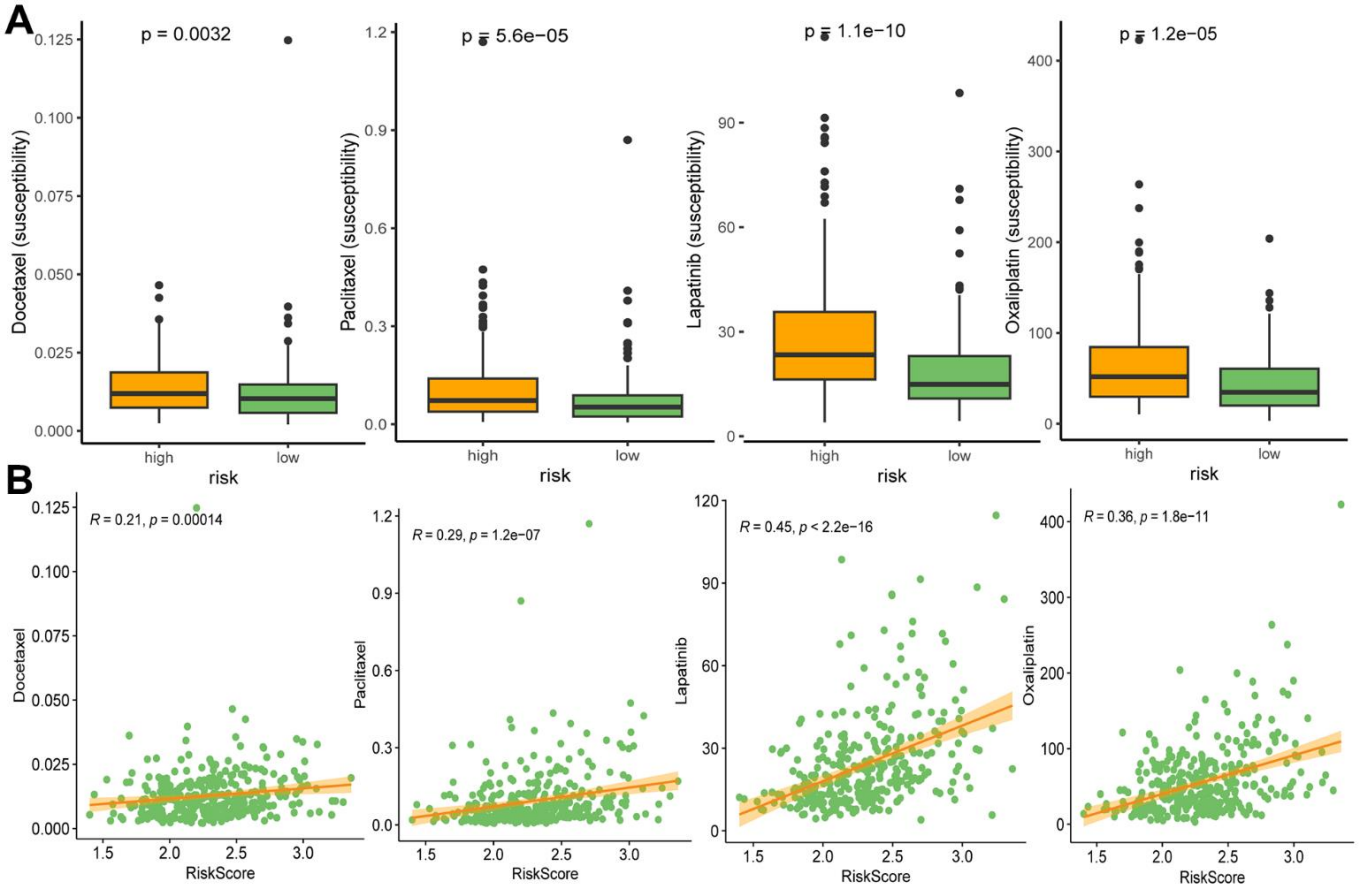
Drug susceptibilities in different risk groups (**A**), and associations between drug susceptibility and risk score (**B**). Docetaxel, paclitaxel, lapatinib, and oxaliplatin are ranked from left to right.

We performed oncoPredict analysis to evaluate the differences in sensitivity of anti-cancer drugs between the high and low risk groups. As a result, four commonly used clinical drugs (including docetaxel, paclitaxel, lapatinib and oxaliplatin) showed distinct sensitivity to different risk groups. All the drugs had better effects on patients in high-risk groups (Figure 10A), and the drug susceptibilities were significantly positively associated with risk scores (Figure 10B).

## DISCUSSION

GC remains a major cancer type with unsatisfactory clinical outcomes, partially due to the failure to optimally utilize the increasingly available targeted and immunological therapies [2]. Current classification methods, such as the conventional AJCC/UICC-TNM system, do not fully leverage recent advancements in personalized therapies, leaving many patients without the most effective treatment options [2]. Over the past decades, numerous new GC classifications have been proposed to improve diagnostic and therapeutic approaches [1, 4]; however, few have been integrated into routine clinical practice. There is a pressing need for sensitive and accurate classification systems that can effectively utilize the variations in the TME composition among patients. These variations are garnering increasing interest as bases for establishing both prognostic tools and therapeutic targets [6, 27]. T cells are the predominant immune cell type within the TME. T_STR_, a unique stress response state of T cells, is associated with genomic, pathological, and clinical parameters in certain cancers and is potentially involved in resistance to anti-PD-L1 immunotherapy [5]. In our study, we pioneered the profiling of T_STR_ cell-related genes in GC tissues compared to normal tissues. This led to a specific focus on CD8+ TSTR cells, which we found to be associated with poor survival outcomes in GC patients. Subsequently, we analyzed CD8+ T_STR_ cell-related DEGs and established an eight-gene signature. This signature proved to be an independent prognostic factor, demonstrating reliability and accuracy in predicting outcomes. It offers potential as a guideline for GC classification, a predictive indicator, and could inform subsequent therapeutic approaches.

TME is increasingly recognized as a critical determinant of cancer characteristics and outcomes. In our study, we systematically examined the biological pathways, immunity, and somatic mutation frequencies in the TME of different risk groups, uncovering several intriguing findings. Notably, our results underscored the vital role of CAFs in patient stratification. CAFs, as the main components and modulators of the extracellular matrix, interact with both cancer cells and cancer-infiltrating immune cells within the TME. This interaction facilitates cancer cell proliferation, therapy resistance, and immune exclusion [28–33]. We observed that extracellular matrix-related events were particularly enriched in the high CD8+ T_STR_ group (Figure 4C). Furthermore, our KEGG analysis indicated significant enrichment of the PI3K-Akt signaling pathway, which is known to induce the transition from pericytes to CAFs [34], in the group with high CD8+ T_STR_ infiltration (Figure 4D). Fibroblasts exhibited a remarkably higher level of infiltration in the TME compared to the other nine major immune cell types (including T cells, CD8+ T cells, cytotoxic lymphocytes, B lineage, NK cells, monocytic lineage, myeloid dendritic cells, neutrophils and endothelial cells) (Figure 8D). Both the infiltration level of CAFs (Figure 8D, 8E) and the TME Stromal score (Figure 8C) were significantly higher in the high-risk group compared to the low-risk group. Among nine TME cell types—CD8+ T, dendritic cells, fibroblasts, gland mucous cells, malignant cells, mast cells, myofibroblasts, pit mucous cells, and plasma cells—fibroblasts had the highest signature risk score, followed by myofibroblasts and malignant cells (Figure 8G). Furthermore, both cancer and inflammatory states exhibit a substantial overlap of extracellular matrix components and share a conserved fibroblast population paradigm [33]. Compared to the low CD8+ T_STR_ group, the high CD8+ T_STR_ group exhibited enrichment in gene hallmarks of inflammatory response, interferon gamma response, and TNF-α signaling via NF-κB (Figure 4B), as well as the cytokine-cytokine receptor interaction pathway (Figure 4D). The inflammatory responses can promote cancer activation, and modulating the inflammatory signaling to enhance cancer sensitivity to immunotherapies has gained benefits for patients in some clinical practice [35, 36]. Therefore, the high inflammatory activation observed may contribute to the poorer outcomes in this group, suggesting that targeting interferon gamma or TNF-α pathways might benefit these patients. Additionally, a distinct difference in somatic mutation profiles between the high and low risk groups could be another crucial factor influencing outcomes. T cells are known to respond to neoepitopes resulting from somatic mutations in cancer cells [37]. Cells with fewer somatic mutations are less likely to present cancer-specific neoepitopes on their surface, leading to a reduced immune response [38]. The high-risk group showed lower frequencies of genetic alterations, particularly in key cancer-related genes such as TTN, TP53, MUC16, and LRP1B.

T_STR_ has been recently identified, and our study delves into this novel realm within the landscape of cancers, offering a nuanced understanding of the T_STR_-related molecular landscape and its implications for GC biology. A key contribution of this work is the identification of T_STR_-related DEGs and their complex associations with GC prognosis. Notably, our exploration of the gene signature in the context of immunotherapy responses and anti-cancer drugs enhances its potential impact on precision medicine for GC patients and informs further experimental studies and clinical trials. Additionally, the functional validation of the signature gene PDGFRL aligns with its prognostic values, expanding our understanding of its biological roles in GC cells. While this T_STR_-related signature underwent validation through experiments and multiple analyses, certain limitations persist. Primarily, it relies on bioinformatics analyses and public data resources. Future work should include prospective studies with larger sample sizes and detailed patient information to enhance the robustness of our findings. High-throughput RNA sequencing of clinical samples collected before and after treatments could further test the predictive power and clinical utility of the signature in real-world settings. Another limitation is the functional validation of the signature gene *PDGFRL*. Given the early stage of research on PDGFRL, its expression levels, detailed biological functions, and underlying mechanisms within GC remain largely unexplored. To address this, future studies could perform co-culture experiments with GC cells and other TME cell types *in vitro* or ideally, utilize an immune-competent GC model to provide deeper insights into PDGFRL functions.

In conclusion, this study marks the first investigation into the molecular characteristics and clinical relevance of T_STR_ in the GC TME, revealing the significant impact of T_STR_ on GC prognosis. We developed and validated a T_STR_ cell-based gene signature, successfully stratifying GC patients into distinct risk groups. The high and low risk groups exhibited notable differences in biological function, mutation status, immunity, and drug susceptibility. This eight-gene signature correlated strongly with OS in GC patients, underscoring its potential clinical utility. Notably, PDGFRL, a key gene within the signature, was found to promote proliferation, migration, and invasion in the GC cell line AGS, further highlighting its role as a risk factor in GC.
